# Identification and Validation of m6A-Related lncRNA Signature as Potential Predictive Biomarkers in Breast Cancer

**DOI:** 10.3389/fonc.2021.745719

**Published:** 2021-10-15

**Authors:** Wenchang Lv, Yichen Wang, Chongru Zhao, Yufang Tan, Mingchen Xiong, Yi Yi, Xiao He, Yuping Ren, Yiping Wu, Qi Zhang

**Affiliations:** Department of Plastic Surgery, Tongji Hospital, Tongji Medical College, Huazhong University of Science and Technology, Wuhan, China

**Keywords:** breast cancer, m6A, lncRNA, prognostic signature, immune infiltration

## Abstract

The metastasis and poor prognosis are still regarded as the main challenge in the clinical treatment of breast cancer (BC). Both N6-methyladenosine (m6A) modification and lncRNAs play vital roles in the carcinogenesis and evolvement of BC. Considering the unknown association of m6A and lncRNAs in BC, this study therefore aims to discern m6A-related lncRNAs and explore their prognostic value in BC patients. Firstly, a total of 6 m6A-related lncRNAs were screened from TCGA database and accordingly constructed a prognostic-predicting model. The BC patients were then divided into high-risk and low-risk groups dependent on the median cutoff of risk score based on this model. Then, the predictive value of this model was validated by the analyses of cox regression, Kaplan-Meier curve, ROC curve, and the biological differences in the two groups were validated by PCA, KEGG, GSEA, immune status as well as *in vitro* assay. Finally, we accordingly constructed a risk prognostic model based on the 6 identified m6A-related lncRNAs, including Z68871.1, AL122010.1, OTUD6B-AS1, AC090948.3, AL138724.1, EGOT. Interestingly, the BC patients were divided into the low-risk and high-risk groups with different prognoses according to the risk score. Notably, the risk score of the model was an excellent independent prognostic factor. In the clinical sample validation, m6A regulatory proteins were differentially expressed in patients with different risks, and the markers of tumor-associated macrophages and m6A regulators were co-localized in high-risk BC tissues. This well-validated risk assessment tool based on the repertoire of these m6A-related genes and m6A-related lncRNAs, is of highly prognosis-predicting ability, and might provide a supplemental screening method for precisely judging BC prognosis.

## Introduction

Breast cancer (BC) represents one of the most fatal malignant tumors prevailed in women, and brings a significant health care burden all over the world. According to the latest cancer epidemiology survey by the American Cancer Society, the incidence of BC continues to increase by about 0.5% every year, and BC alone will account for 30% of newly diagnosed cancers for women in 2021 (1). In recent years, despite the progress of effective therapeutic strategies, including surgery, chemotherapy, hormone therapy, and immunotherapy, BC is still confronted with high incidence rates, high invasion, metastasis, and relapse rate (2). Notably, nearly 20-30% of BC patients may have metastasis after diagnosis and treatment of the primary tumor and about 90% of BC-related deaths are attributed to metastasis (3). More importantly, it is well-documented that BC is a complex tumor type with high genetic heterogeneity, and different BC subtypes display significant biological characteristics and different activities in response to the regimen. Studies have emphasized that the BC signatures with malignant molecular phenotype are more prone to the undesirable prognosis of BC (4–7). Specific molecular biomarkers are pivotal clues for the early detection and intervention of BC. Therefore, the establishment of the prognostic risk model could guide the screening and identification of high-risk patients and might help improve clinical outcomes.

N6-methyladenosine (m6A) is the most common modification of mRNA in mammals, possessing a complex and fine-tuned regulatory system that dynamically and reversibly modulates splicing, localization, transport, translation, and stability of mRNA (8, 9). There are three main regulator proteins that mediate m6A modification, including methyltransferase (writer), demethylase (eraser), and binding protein (reader) (10, 11). Besides, cumulative evidence indicates that m6A modification plays an essential role in multiple biological processes involved in determining cell fate. Especially, the aberrant mA modification has recently been intensively involved in carcinogenesis in various cancers. These m6A regulators profoundly affect the development and progression of BC, possessing the value of being early diagnostic, prognostic biomarkers, as well as therapeutic targets.

Currently, m6A modifications have also been found in non-coding RNAs, including long non-coding RNAs (lncRNAs), microRNAs (miRNAs), and small nuclear RNAs (snRNAs). Among them, lncRNA is defined as non-coding RNA with more than 200 nucleotides in length, which participates in chromatin interaction, transcriptional regulation, RNA processing, mRNA stability, translation, and cell signal transduction (12, 13). Recently, various studies have identified a lot of differently expressed lncRNAs in BC and have been reported to be crucial orchestrators in the multistep process of BC tumorigenesis and malignant transformation. The m6A modification and lncRNA may play synergetic roles in tumorigenesis and development through a variety of regulatory mechanisms (9, 12, 14, 15). For example, the m6A mediated by METTL3 was found to conduce to increase the expression of LINC00958, thereby aggravating the malignant phenotype of hepatocellular carcinoma (16). Besides, Wu et al. also illustrated that ALKBH5-mediated up-regulation of lncRNA PVT1 could promote the growth and proliferation of osteosarcoma cells *in vitro* (17). Moreover, the lncRNA RP11 modified by m6A was found to regulate Fbxo45/Zeb1 which might trigger the occurrence and development of colorectal cancer (18). However, the precise functional details of m6A regulators and lncRNA, as well as their congenerous contribution to the occurrence and clinical outcome of BC, are yet to be fully elucidated.

**Figure 1:**
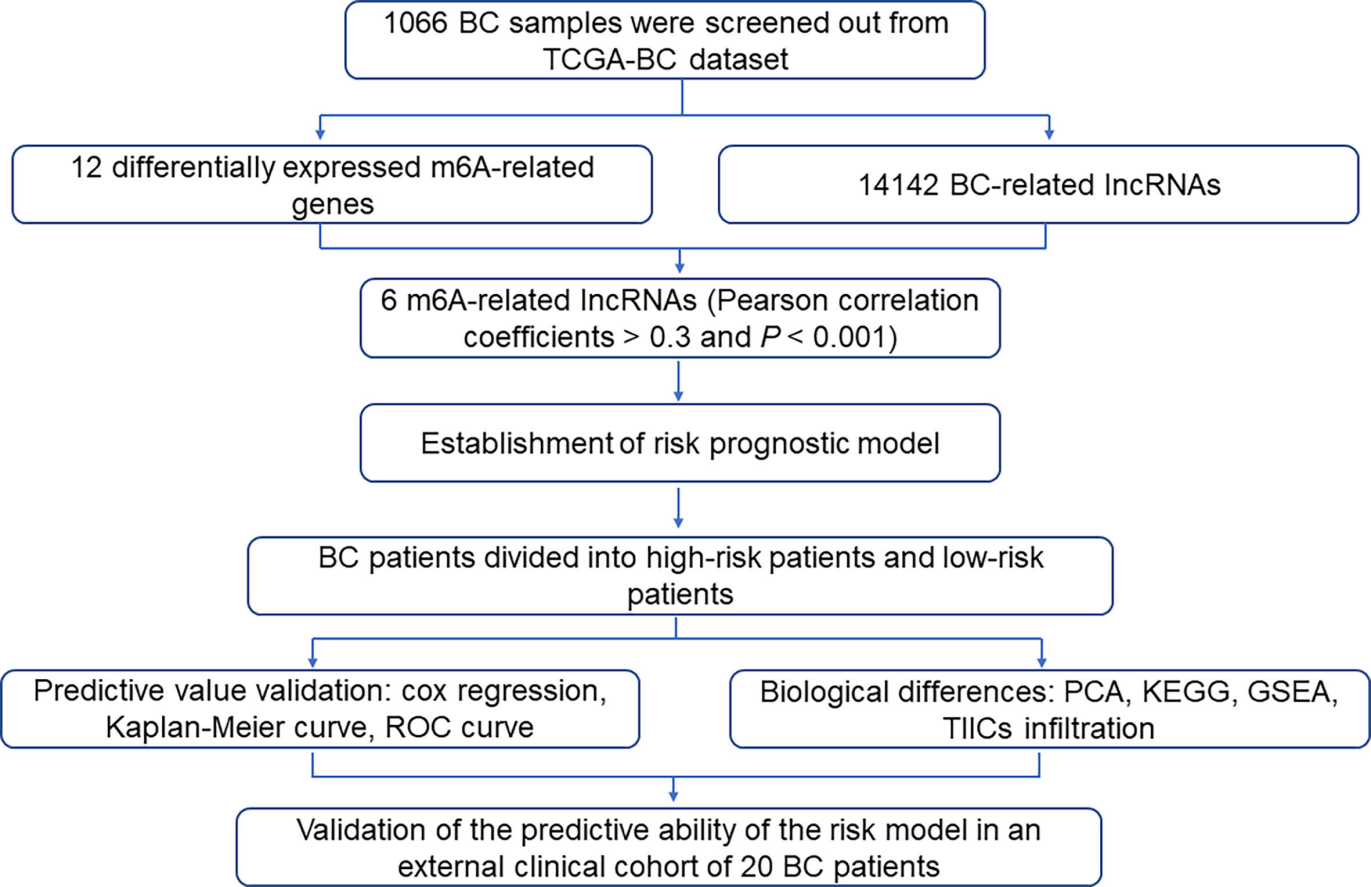
Flowchart showed procedures involved in this study.

Therefore, understanding the potential link of m6A modification and lncRNA involved in BC may contribute to the establishment of a prognosis-predicting system for BC. In the present, we firstly identified 6 m6A-related lncRNAs as having prognostic values in BC patients in The Cancer Genome Atlas (TCGA) database. Then, we accordingly constructed a risk prognostic model based on the ability of 6 m6A-related lncRNAs to predict the overall survival (OS) of BC patients. More importantly, according to the risk score, the BC patients were successfully divided into the low-risk and high-risk groups. The two groups not only possessed different BC prognoses, but also showed different gene expression profiles and different TILs characteristics. Besides, the M2 macrophage markers and m6A regulatory proteins were co-expressed in high-risk BC tissues, and m6A regulatory proteins were differentially expressed in patients with different risks. **Figure 1** showed a flowchart of procedures involved in this study. Targeting the repertoire of these m6A-related genes and m6A-related lncRNA might provide more guiding significance for the prognosis and even precise therapy of BC. This well-validated risk assessment tool in this study might provide a supplemental screening method for precisely judging BC prognosis.

## Materials and Methods

### Information Acquisition of BC Patients

RNA transcriptome profiling data and corresponding clinical information of BC patients were acquired from TCGA database (https://cancergenome.nih.gov/). Ultimately, a total of 1178 patients were enrolled in this study, including 1066 tumor samples and 112 normal samples, and the detailed clinical information of included BC patients was collected in **Supplementary Table 1**. A total of 20 patients who had undergone breast cancer were enrolled in the external valid cohort, the detailed clinical information of included BC patients was presented in **Supplementary Table 2**.

### Identification of m6A-Related Genes and m6A-Related lncRNAs

We extracted the gene expression data of m6A RNA methylation regulators from the TCGA database, including the following genes: writer: METTL3, METTL14, WTAP, ZC3H13, RBM15B, RBM15, KIAA1429; eraser: ALKBH3, ALKBH5, FTO; reader: YTHDC1, YTHDC2, YTHDF1, YTHDF2, YTHDF3, HNRNPA2B1, HNRNPC. They were defined as m6A-related genes. Then we identified 14142 BC-related lncRNAs in the TCGA database based on the lncRNA annotation file. The Pearson correlation analysis was carried out to analyze the expression level of m6A-related genes and annotated lncRNAs. The lncRNAs with |Pearson R| > 0.3 and *P* value < 0.001 were referred to as m6A-related lncRNAs and considered for subsequent analysis.

### Principal Component Analysis (PCA) and Gene Set Enrichment Analysis (GSEA)

The PCA analysis was used to reduce the dimensionality of large gene expression data and perform hierarchical clustering on all samples. Besides, the distribution of all samples was visualized by 3D scatter diagram. Moreover, to explore the difference in the underlying molecular signaling mechanisms between the low-risk and high-risk groups, the GSEA was performed to investigate enriched items. The false discovery rate (FDR) < 25% and *P* adjusted value < 0.05 was selected as statistically significant criteria.

### Nomogram Construction

The independent predictors determined by multivariate Cox regression analysis were included to establish a nomogram model, aiming at evaluating the predictive power of independent predictors for 1-year, 3-year, and 5-year OS rates. Subsequently, a calibration plot was established to calculate the consistency index to evaluate the accuracy of the prediction ability of the nomogram.

### Establishment of Risk Score and Construction of Predictive Risk Model

Lasso and multivariate cox regression were further used to screen the significant lncRNAs and determine the coefficients to construct the risk model. The risk-score formula was defined as follows: Risk score = *Coe_1_* *Exp_1_+ Coe_2_ *Exp_2_+ Coe_3_ *Exp_3_+……+ Coe_n_ *Exp_n_. *Coe* was the coefficient of the multiple Cox regression analysis of the 6 lncRNAs, and *Exp* was the corresponding expression value. According to the median risk score, the patients were classified into the high-risk group and low-risk group.

### Quantitative Real-Time PCR (qRT-PCR)

Total RNA of BC tissues was extracted by trizol (Takara, Japan) according to the manufacturer’s protocols and used for the synthesis of cDNA with the 1st Strand cDNA Synthesis Kit (Yeasen, China). Duplicate samples for qRT-PCR were carried out using SYBR GreenTM Master Mix (Yeasen, China) and performed with QuantStudio1 (ABI Q1, USA). Primer sequences can be obtained in **Supplementary Table 3**.

### Immunohistochemistry

For immunohistochemistry, the breast sections were rehydrated by different concentrations of alcohol and were retrieved by heating slides in citrate buffer using pressure cooker. We immune-stained sections with primary antibodies against human METTL3 and human METTL14 (all 1:100, Proteintech, China) overnight at 4°C followed by horseradish peroxidase (HRP) conjugated secondary antibodies, which was visualized using a DAB Peroxidase Substrate Kit (Maxin, China) and counterstained with Hematoxylin. Digital images of the sections were captured by a SOPTOP CX40 microscope (China).

### Immunofluorescence

For immunofluorescence, the breast sections were processed the same as immunofluorescence before staining. Concisely, sections were incubated at 4°C overnight with a cocktail of primary antibodies diluted in antibody diluent, at the following dilutions: anti-human METTL3 and anti-human METTL14 (1:100, Proteintech, China). The following day, the primary antibodies were detected by incubation of a cocktail of secondary antibodies (Life Technologies, USA) for 1 h at room temperature. The slides were counterstained with nuclear 4,6-diamidino-2-phenylindole (DAPI, Vector Laboratories; Burlingame). Olympus fluorescence microscope (Japan) was used to capture images.

### Statistics Analysis

The Kaplan-Meier curve was used to present the OS rate, and the two-sided log-rank test was used to evaluate the OS between different groups. Univariate and multivariate Cox regression analyses were performed to verify the independent prognostic factors for BC, hazard ratios (HR) and 95 % confidence intervals (CI) were displayed. The area under the ROC curve (AUC) calculated from the ROC curve analysis was used to assess the prognostic performance of the lncRNA risk score. The Wilcoxon test examined the differences for variables of two groups. Two-sided *P* value < 0.05 was considered significant. The R language R x64 4.0.5 was used for all statistical analyses.

## Results

### Altered Expression of the m6A RNA Methylation Regulators in BC

**Figure 2:**
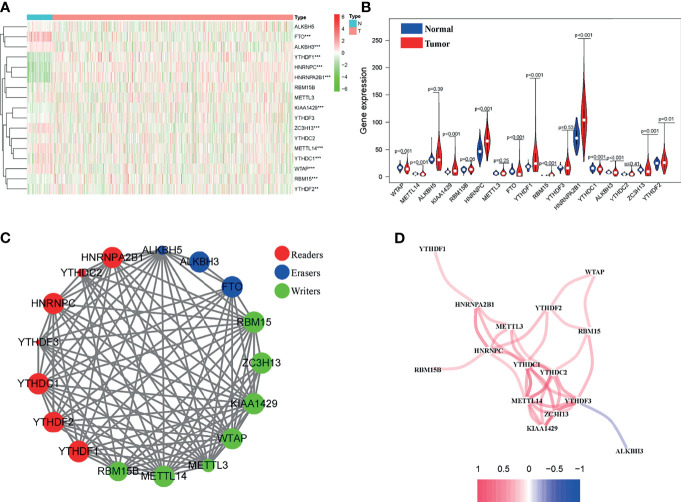
Identification of m6A-related genes in BC patients. **(A)** Heatmap of 17 m6A-related genes in BC tissues and normal tissues from the TCGA database. **(B)** Violin plot of 12 differentially expressed m6A regulator genes. **(C)** Gene interaction network map of three types of m6A regulator genes in BC patients. **(D)** The positive and negative correlation between m6A-related genes in BC patients. *P < 0.05, **P < 0.01, ***P < 0.001.

The abnormal level of m6A modification caused by m6A modulators is emerging as a common characteristic of various tumors. Hence, to decipher the potential link of m6A modulators and BC, we analyzed the mRNA expression level of 17 m6A-related genes, including writer, eraser, and reader in a total of 1066 BC tissues and 112 normal tissues from the TCGA database, which showed that 12 out of the 17 m6A-related genes were differentially expressed in BC tissues compared to normal tissues (**Figure 2A**). Among these 12 aberrantly expressed m6A regulator genes, KIAA1429, HNRNPC, YTHDF1, RBM15, HNRNPA2B1, YTHDF2 were significantly up-regulated, while WTAP, METTL14, FTO, YTHDF3, YTHDC1, ALKBH5, and ZC3H13 exhibited decreased expression in BC tissues (**Figure 2B**). The results of **Figures 2C, D** showed strong interactions between the 17 m6A-related genes in BC. The above results indicated that this group of m6A-related genes might be involved in the tumorigenesis and progression of BC.

### Identification of Differentially Expressed m6A-Related lncRNAs in BC Patients

**Figure 3:**
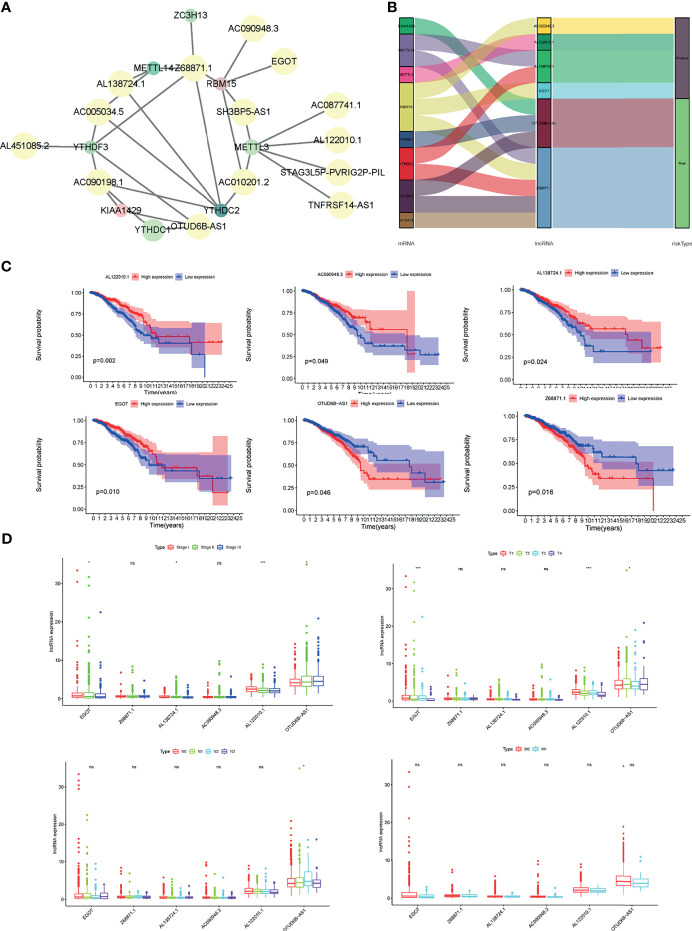
Identification of m6A-related lncRNAs in BC patients. **(A)** Correlations between m6A-related genes and 6 m6A-related lncRNAs. **(B)** Sankey relational diagram for 17 m6A genes and m6A-related lncRNAs. **(C)** Kaplan-Meier survival curves of 6 candidate m6A-related lncRNAs in the BC cohort. **(D)** Expression levels of m6A-related lncRNAs with tumor stage, and TNM classification of BC patients. *P < 0.05, ***P < 0.001. ns, no significance.

Then, we obtained the annotated files of lncRNA and identified 14142 BC-related lncRNAs in the TCGA data set. The lncRNAs, whose expression values were associated with one or more of the 12 m6A-related differentially expressed genes (Pearson correlation coefficients > 0.3 and *P* < 0.001), were defined as m6A-related lncRNAs. Under the criteria, a total of 6 lncRNAs closely related to m6A regulator genes were screened out, namely Z68871.1, AL122010.1, OTUD6B-AS1, AC090948.3, AL138724.1, EGOT (**Figure 3A**). Next, the univariate Cox regression analysis was conducted to explore their values in evaluating the prognosis of BC patients. The results demonstrated that among the 6 m6A-related lncRNAs, AL122010.1, AC090948.3, AL138724.1, and EGOT were considered as protective factors for BC, while Z68871.1 and OTUD6B-AS1 were regarded as risk factors (**Figure 3B**). Further, the Kaplan-Meier survival curves of the 6 candidate m6A-related lncRNAs were delineated in the BC patients, which suggested that the high expressions of AL122010.1, AC090948.3, AL138724.1, and EGOT were associated with better OS rates. Conversely, the low expression of Z68871.1 and OTUD6B-AS1 were correlated with better OS rates for BC patients (**Figure 3C**). In addition, the expression levels of m6A-related lncRNAs were found concerning the clinical characteristics of BC patients, including patient tumor stage, and TNM classification (**Figure 3D**).

### Identification of Prognostic m6A-Related lncRNAs and Establishment of Risk Models

**Figure 4:**
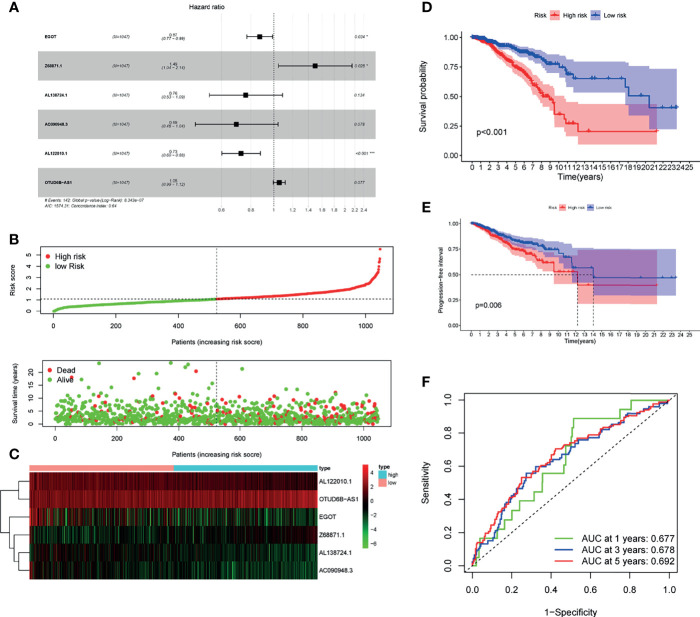
Risk model for BC patients based on m6A-related lncRNAs. **(A)** LASSO regression analysis was performed on the 6 m6A-related lncRNAs. **(B)** Multivariate Cox regression analysis revealed the correlation of clinical prognosis and candidate m6A-related lncRNAs. **(C)** Distributions of m6A-related lncRNA model-based risk score and survival status of BC patients in the TCGA dataset. **(D)** Expression of the 6 prognostic lncRNAs for each patient in clustering analysis. **(E)** Kaplan-Meier curves of patients assigned to high- and low-risk groups. **(F)** Time-dependent ROC curves and AUC showed predictive efficiency of risk scores and other clinical characteristics. *P < 0.05, ***P < 0.001.

Next, to establish a risk predictive model, the multivariate Cox regression analysis was performed on the 6 m6A-related lncRNAs that were previously identified in the TCGA database (**Figure 4A**). The multivariate Cox regression analysis suggested that the total 6 m6A-related lncRNAs were all independent prognostic factors for BC. Besides, AL122010.1, AC090948.3, AL138724.1, and EGOT were considered to be protective factors for BC [(HR) < 1], while Z68871.1 and OTUD6B-AS1 were risk factors for BC (HR > 1). Consequently, we constructed a risk score based on the coefficient from multivariate Cox regression and the expression of each lncRNA, and ultimately divided the BC patients into the low-risk group and high-risk group according to the median risk score (**Figure 4B**). The 6 m6A-related lncRNAs had distinctly different expression patterns in the low-risk group and high-risk group (**Figure 4C**). Patients in the high-risk group had a significantly lower OS rate and a shorter OS duration than those in the low-risk group (**Figure 4D**). Also, Patients in the high-risk group had a significantly lower PFI (progression free interval) rate and a shorter PFI than those in the low-risk group (**Figure 4E**). Hence the risk score had prognostic value for tumor progression or relapse. It was consistent that according to the ROC curve, the m6A-related lncRNAs were of value to predict the OS rate in the BC cohort, accompanied with the 1-year AUC = 0.677, 3-year AUC = 0.678, and 5-year AUC = 0.692 (**Figure 4F**).

### Prognostic Value of the Risk Models Based on 6 m6A-Related lncRNAs in BC Cohort

**Figure 5:**
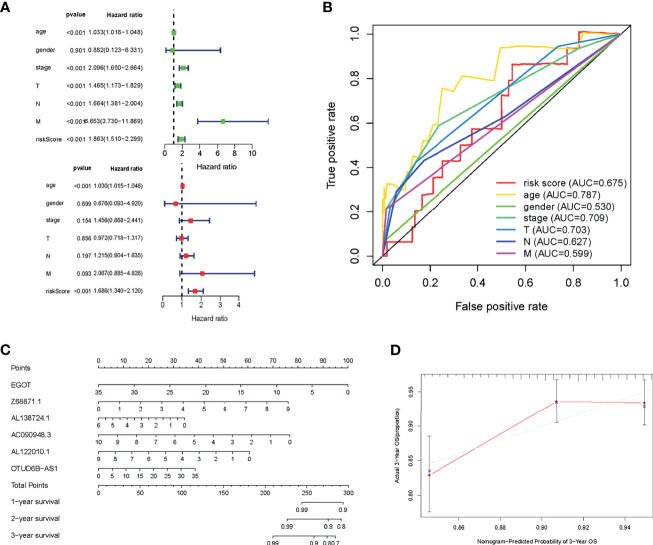
Prognostic value of the risk model of the 6 m6A-related lncRNAs. **(A)** Forest plot for univariate and multivariate Cox regression analysis of risk score and other clinical characteristics. **(B)** ROC curves for the nomogram, risk score, age, gender, grade, and TNM in the TCGA dataset. **(C)** Nomogram model based on the 6 m6A-related lncRNAs predicted the probability of OS at 1, 3, and 5 years in the TCGA dataset. **(D)** Calibration plots of the nomogram for internal validation.

Subsequently, in combination with the age, gender, tumor stage, and TNM classification of BC patients, the Cox regression was used to verify whether the risk score was an independent prognostic factor for BC patients. The univariate Cox regression showed that the HR of risk score was 1.863 (**Figure 5A**), and multivariate Cox regression showed that the HR of the risk score was 1.686 (**Figure 5B**). The predictive potency of the risk score was further confirmed by the ROC curve, with the AUC of the risk score was as high as 0.675 (**Figure 5C**). These 6 m6A-related lncRNAs were put into the Nomogram model to predict the OS of BC patients at 1, 3, and 5 years, and the calibration curve demonstrated that lncRNAs possessed an ideal consistency in predicting the 3-year OS rate of BC patients (**Figures 5D, E**).

### Different Gene Expression Profiles Between the Low-Risk Group and High-Risk Group

**Figure 6:**
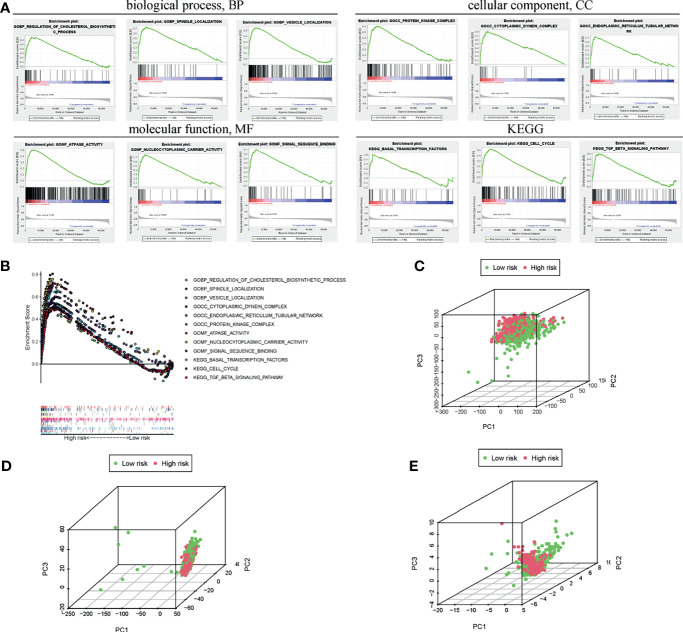
Functional analysis of pathway and process enrichment between the high- and low-risk groups. **(A)** GSEA indicated the enrichment of various biological processes in the high-risk subgroup. **(B)** Enrichment scores of the above processes were collectively exhibited. PCA between the high- and low-risk groups based on the transcriptome **(C)**, BC-related lncRNAs **(D)**, and m6A-related lncRNAs **(E)**.

To further clarify the specific molecular differences between low-risk and high-risk subgroups, 56754 differentially expressed genes were identified and functional annotation was performed by the GSEA. The differentially expressed genes were mainly clustered in multiple important pathways, including Biological processes: regulating cholesterol biosynthesis, spindle localization, vesicle localization; Cell components: protein kinase complex, cytoplasmic dynamic protein complex, endoplasmic reticulum tubular network; Molecular functions: ATPase activity, nucleocytoplasmic carrier activity, signal sequence binding; KEGG: basic transcription factor, cell cycle, and TGF-β signaling pathway (**Figure 6A**). Moreover, the GSEA also revealed that activities related to these biological processes were more frequent in the high-risk group (**Figure 6A**). The enrichment scores of the above process were together exhibited in **Figure 6B**. Furthermore, the PCA results revealed different whole transcriptome expression pattern (**Figure 6C**), BC-related lncRNA expression pattern (**Figure 6D**), and m6A-related lncRNA expression pattern between the low-risk and high-risk group (**Figure 6E**). Based on the expression profile of m6A-related lncRNA, the high-risk group are more separated from the low-risk group.

### Correlation Between Tumor-Infiltrating Immune Cells (TIICs) and m6A-Related lncRNAs in BC Patients

**Figure 7:**
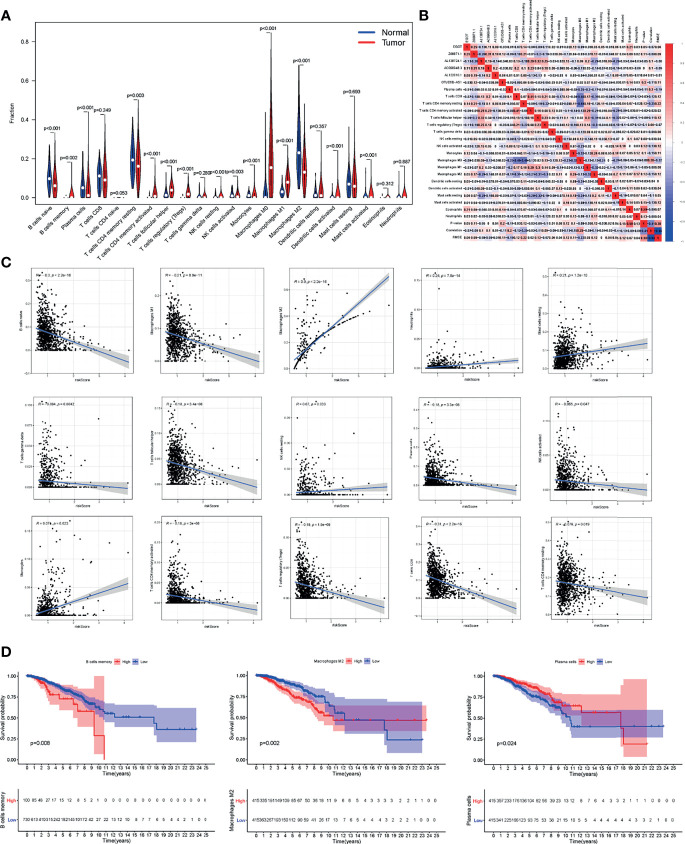
The m6A-related lncRNAs in BC were related to the TIICs. **(A)** The abundance of TIICs in tumor tissues and normal tissues. **(B)** Heatmap showed the relationship between TIICs and the 6 m6A-related lncRNAs. **(C)** Pearson correlation between risk score and differentially infiltrated TIICs. **(D)** Kaplan-Meier curves of patients with differentially infiltrated memory B cells, M2 macrophage, and plasma cells.

Interestingly, here we quantified immune cells to further explore the infiltration ratio of different TIICs in BC tissue. The infiltration fraction in BC tissues and normal tissues was displayed in **Figure 7A**, and 15 out of 22 types of immune cells all showed obvious differences in infiltration. The TIICs within breast tumors were proposed to impact prognosis and determine clinical outcomes, so we attempt to explore the connection between TIICs and our risk model. The complex correlations between multiple TIICs and 6 m6A-related lncRNAs could be seen in **Figure 7B**. Among all TIICs with different infiltration ratios, M2 macrophages, neutrophils, mast cells, NK cells, and monocytes were positively correlated with the aforementioned risk scores, while other types of TIICs were in negative correlation (**Figure 7C**). In the OS analysis of BC patients, low abundance of memory B cells and M2 macrophages as well as the high abundance of plasma cells, were associated with better OS rates of BC patients (**Figure 7D**). Totally, these results highlighted the association between m6A-related lncRNAs and TIICs, indicating the potential immune-modulating role of the m6A-related lncRNAs.

### Association Between TIICs and m6A-Related Genes

**Figure 8:**
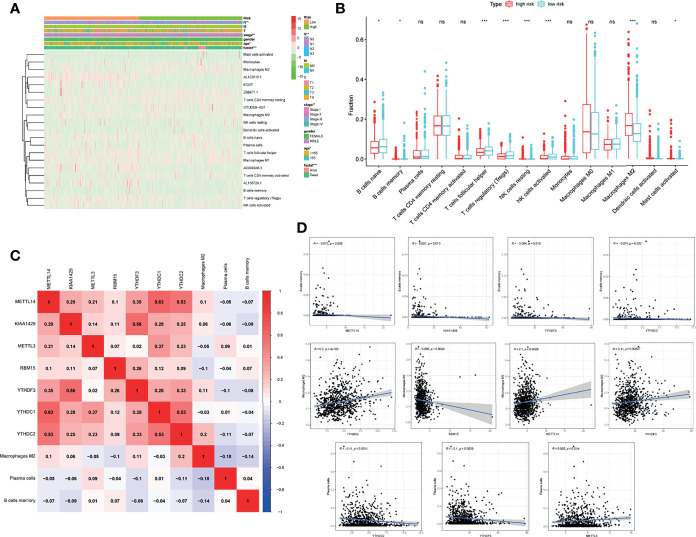
The m6A-related genes in BC were related to the TIICs. **(A)** Expression of m6A-related lncRNAs and TIICs together with clinicopathologic features including node, stage, age, and status in each patient was shown in the heatmap. **(B)** 15 TIICs abundance in high-risk and low-risk groups. **(C)** Heatmap showing the relationship between m6A-related genes and TIICs. **(D)** Pearson correlation between m6A-related genes and memory B cells, M2 macrophage, and plasma cells. *P < 0.05, **P < 0.01, ***P < 0.001. ns, no significance.

After dividing the included patients into high-risk and low-risk groups using risk scores, a heatmap about the distribution of clinicopathologic features was plotted, and the evidential significance of node (*P* < 0.01), stage (*P* < 0.01), age (*P* < 0.05), and status (*P* < 0.001) were observed between high-risk and low-risk groups (**Figure 8A**). Expression of m6A-related lncRNAs and TIICs were also calculated in each patient and shown in the heatmap. About 9 of the 15 different TIICs showed risk-related differences in abundance in the two groups (**Figure 8A**). The abundance of M2 macrophages in the high-risk group was significantly higher than that in the low-risk group, while the abundance of naive B cells, memory B cells, follicular T helper cells, regulatory T cells (Tregs), NK cells, and activated mast cells were declined in the high-risk group (**Figure 8B**). The detailed association between m6A-related genes and abnormally infiltrated TIICs was displayed in **Figure 8C**. Intriguingly, the memory B cells were found to be negatively correlated with METTL14, KIAA1429, YTHDF3 and YTHDC2. However, M2 macrophage was positively correlated with YTHDC2 and METTL14, but negatively correlated with RBM15 and YTHDF3. Besides, the plasma cells were positively related to METLL3 and negatively associated with YTHDC2 and YTHDF3 (**Figure 8D**).

### Validation of the Predictive Ability of the Risk Model in an External Clinical Cohort

**Figure 9:**
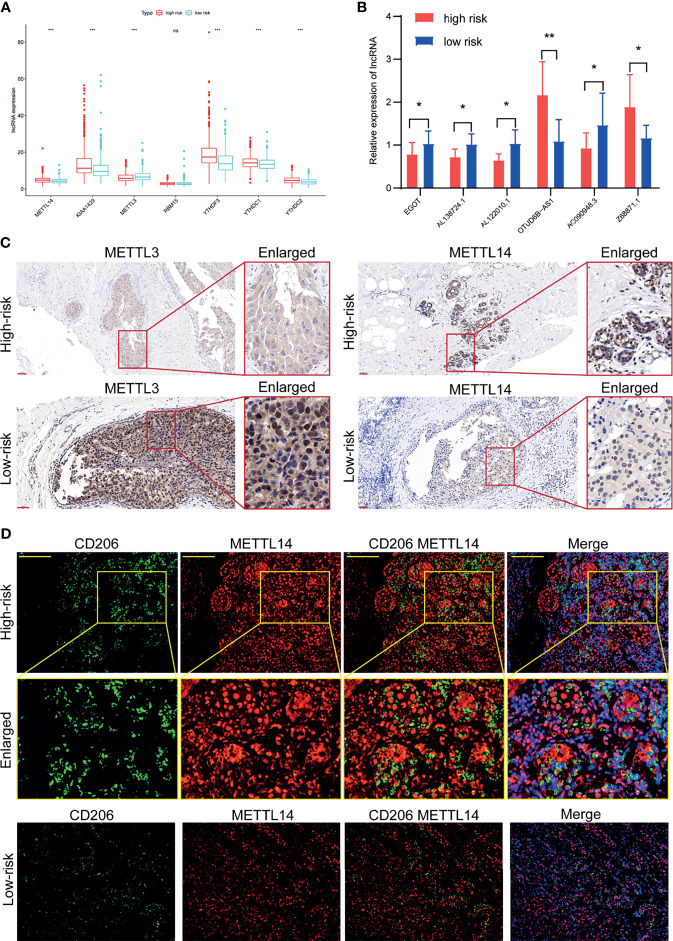
Validation of the association between m6A regulators and tumor microenvironment in a clinical cohort. **(A)** Expression of m6A regulators in high-risk group and low-risk group. **(B)** Expression of the 6 m6A-related lncRNAs in our clinical validation cohort. **(C)** The expression levels of METTL3 were significantly increased and METTL14 were significantly decreased in the high-risk patients than those in the low-risk patients of the clinical validation cohort. **(D)** Abundant M2 macrophage infiltration and co-expression of M2 macrophage marker CD206 and METTL14 was found in high-risk patients of the clinical validation cohort. *P < 0.05, **P < 0.01, ***P < 0.001. ns, no significance.

A clinical cohort of 20 BC patients with different stages was established to validate the correlation between m6A-related genes, m6A-related lncRNAs, and TIICs. Firstly, the m6A regulators including METTL14, KIAA1429, METTL3, YTHDF3, YTHDC1, and YTHDC2 were observed remarkably differently expressed between the high-risk group and low-risk group (**Figure 9A**). So, the METTL3 and METTL14 were used to verify patients with different risks and CD206 was used to represent the relative abundance of M2 macrophage. Then, the relative expressions of the 6 m6A-related lncRNAs in the 20 BC patients were analyzed by qRT-PCR (**Figure 9B**). Afterward, the risk score of each patient was calculated according to the formula (Risk score = Coef1*Exp1+ Coef2*Exp2+ Coef3*Exp3+……+ Coefn*Expn, n=6). Among them, *Coef* was derived from the coefficient of multi-Cox regression of BC patients in the TCGA (the *Coef* of EGOT was -0.136, the *Coef* of Z68871.1 was 0.401, the *Coef* of AL138724.1 was -0.273, the *Coef* of AC090948.3 was -0.365, the *Coef* of AL122010.1 was -0.319, and the *Coef* of OTUD6B-AS1 was 0.052), but the *Exp* was the expression of m6A-lncRNA results of qPCR. The risk scores of each patient (n=20) were calculated based on the above formula. Accordingly, we divided the 20 BC patients into the high-risk group and low-risk group. The IHC results confirmed the declined expression of METTL3 but the elevated expression of METTL14 in high-risk patients in comparison to the low-risk patients (**Figure 9C**). The further IF assay indicated that the M2 macrophages were more abundant in high-risk patients, and the M2 macrophage marker CD206 and m6A regulator protein METTL14 were found to more co-express in the high-risk patients (**Figure 9D**).

## Discussion

Clinically, pathological staging is still the most important guideline for the diagnosis and treatment of BC. But, it was surprising that even patients with the same stage of BC often vary greatly in prognosis, strongly implying that the current staging systems are not entirely accurate to provide survival predictions and reflect BC heterogeneity. Up to date, emerging studies have attempted to construct novel and effective strategies to address the deficiencies of accuracy and precision. The tentative methods includes the risk model based on tumor-specific ncRNAs patterns/signatures, the exosome-based tumor recognition, the immune infiltration characteristics, the genome-wide expression and somatic mutation analysis (19, 20). These methods could serve as complementary or alternative prediction platforms with the conventional prediction method. In recent years, lncRNAs are novel, potential therapeutic targets and biomarkers for cancer treatments. Scholars have devoted themselves to elucidating the downstream mechanism of differential expressed lncRNAs in tumors, but lncRNA upstream regulatory mechanism has not attracted much attention. Few reports have been related to these aspects in BC, especially the upstream regulatory mechanisms of m6A modification in lncRNAs. Therefore, focusing on the interaction of m6A modification and lncRNAs will help better understanding their roles in BC and can identify potential prognostic markers or therapeutic targets of BC.

The present study included 1066 BC patients from the TCGA dataset to explore the prognostic significance of m6A-related lncRNA in BC. After identifying the correlation between the 6 m6A-related lncRNAs and clinical features, the univariate and multivariate Cox regression analysis confirmed that these lncRNAs had independent prognostic value for BC patients. Importantly, these screened 6 m6A-related lncRNAs were used to establish a risk score model to predict OS in BC patients, which was able to successfully and effectively divide patients into the high-risk group and low-risk group. The further analysis also proved that the risk score established by the m6A-related lncRNA model was an independent risk factor for BC patients. Conformably, the ROC curve and the nomogram model further verified the prognostic accuracy of the risk score. Then, the GSEA was used to investigate the differences in biological behaviors between the high-risk group and the low-risk group, showing that the activities related to the cell cycle, TGF-β signaling pathway, and other processes were more frequent in the high-risk group. Subsequently, we evaluated the difference in TIICs between the high-risk group and the low-risk group. The results demonstrated that the high-risk group had a higher infiltration abundance of M2 macrophages, neutrophils, resting mast cells, resting NK cells, and monocytes. Using a clinical validation cohort, it provided further evidence on the predictive power of the risk model. The high-risk patients had different expression levels of METTL3 and METTL14 from the low-risk patients. The high infiltration of M2 macrophages in high-risk patients again verified the poor prognosis in high-risk BC patients. Hence, the successfully established m6A-related lncRNA model provides a new and effective method for predicting the prognosis of BC patients. In this risk prediction model, the higher the risk score, the worse the prognosis of high-risk groups, and vice versa. It provides a train of thought for further research on the diagnostic value of m6A-related lncRNAs.

Currently, many studies have proved that m6A modification may coordinate and orchestrate the onset and progression of various cancers. Intriguingly, the m6A modulator can mediate or maintain the malignancy tumors by modifying specific lncRNAs. For example, lncRNA GATA3-AS was previously reported that could promote the m6A modification of GATA3 precursor mRNA mediated by the writer protein KIAA1429, thus promoting the growth and metastasis of liver cancer *in vivo* (21). In a glioma research, the lncRNA FOXM1-AS exerted the function to facilitate glioblastoma stem cell-like cells proliferation by enhancing the interaction of ALKBH5-FOXM1 (22). Moreover, lncRNA GAS5-AS1 had been identified as a promoter in ALKBH5-dependent m6A demethylation in cervical cancer, consequently inhibiting proliferation, migration, and invasion of cervical cancer cells (23). The above studies have illustrated that the interaction between m6A and lncRNA can affect the tumorigenesis and progression of cancer. Exploring the role of m6A-modified lncRNA in tumors will provide a broad prospect for early detection, prevention, and treatment of tumors. Several identified m6A-related lncRNAs in this study had been reported to relate to BC prognosis, which was consistent with our results. For instance, AL122010.1 was considered to be a lncRNA related to BC stem cells (24), autophagy (25), and immunity (26), and performed as a biomarker for risk prediction in BC patients. OTUD6B-AS1 could be used as a survival prognosis factor for BC (26). And two independent studies both suggested that Z68871.1 had prognostic value in BC patients (24, 27). It was also reported that EGOT could be used as a BC survival predictor (28). Most studies only focus on a single molecule, but the development and metastasis of tumors are often the results of the synergy of multiple molecules. In the present study, we identified 6 prognostic m6A-related lncRNAs in BC and build a risk model based on the 6 m6A-related lncRNAs together, this will provide more comprehensive predictive information than a single molecule.

TME is a complex ecosystem composed of cancer cells, immune cells, fibroblasts, and vascular cells. It is widely acknowledged that the immune cells which interact with BC could impact patient prognosis and determine clinical outcomes (29). More importantly, previous studies have confirmed that M2 macrophages secrete protumoral cytokines to facilitate tumor proliferation, angiogenesis, metastasis, modulation of extracellular matrix, thus leading to unfavorable clinicopathological features and poor prognosis in BC (30). Our findings were accordant with the above conception that the high-risk group had a higher infiltration abundance of M2 macrophages concomitant with co-expression of M2 macrophages markers and m6A regulators in the high-risk patients. This evidence supports the predictive ability of our risk prognostic model.

Collectively, the current study introduced a repertoire of m6A-related lncRNAs as a novel prognostic tool for BC patients. This study is characterized by the establishment of a model based on a comprehensive population database and high-throughput sequencing data, which was successfully validated by subsequent BC tissue sample detection in an external clinical cohort. Nevertheless, there are still some concerns that need to be mentioned for the potential clinical translational application. In the first place, since this study is actually a retrospective study and the analysis data are from the open-access online databases and sample validation, the prediction effect of this model in practice needs to be verified in collaboration with conventional prediction methods in the future. Secondly, as different bioinformatics algorithms may lead to different results, additional quantities of external verification of other clinical datasets would be beneficial to decipher the predictive role of m6A-related lncRNAs in BC more extensively. Third, we would like to emphasize that the risk score was a supplement rather than a replacement. The results just showed that our prediction model can add predicting value to the TNM system. The combination of the risk score, the TNM system, and age synergistically or complementally, was very necessary for clinical work. Ultimately, although we preliminarily explored expression signature and the immune link of m6A-related lncRNA, these lncRNAs were not yet fully elucidated and are worth further in-depth investigation. In the following work, we will continue to verify the prognostic accuracy of the model on a large scale with more samples and more external experiments.

## Conclusion

In summary, we successfully constructed a prognostic model containing 6 m6A-related lncRNAs in the present study. According to the risk score of this model, the high-risk BC patients exhibited the expected worse clinical outcomes, and vice versa. This well-validated risk assessment tool based on the repertoire of these m6A-related lncRNAs, is of highly prognosis-predicting ability for BC. Further studies exploring the m6A-related lncRNAs and their biological functions will endow huge potentials in developing therapeutic strategies for combating BC.

## Data Availability Statement

The datasets presented in this study can be found in online repositories. The names of the repository/repositories can be found in the article/**Supplementary Material**.

## Ethics Statement

The studies involving human participants were reviewed and approved by Medical Ethics Committee of Tongji Hospital, Tongji Medical College, Huazhong University of Science and Technology. The patients/participants provided their written informed consent to participate in this study.

## Author Contributions

WCL and YCW had an equal contribution to this manuscript. QZ designed the whole study. WCL and YCW participated in the bioinformatics and statistical analysis, CRZ and YFT did the immuno-histochemistry analysis. MCX performed real-time PCR analysis. YY and XH made the manuscript and figure editing. YPR and YPW revised the manuscript. All authors contributed to the article and approved the submitted version. 

## Funding

This article acknowledges contributions from the China GuangHua Science and Technology Foundation (No. 2019 JZXM001) and Wuhan Science and Technology Bureau (No. 2020020601012241).

## Conflict of Interest

The authors declare that the research was conducted in the absence of any commercial or financial relationships that could be construed as a potential conflict of interest.

## Publisher’s Note

All claims expressed in this article are solely those of the authors and do not necessarily represent those of their affiliated organizations, or those of the publisher, the editors and the reviewers. Any product that may be evaluated in this article, or claim that may be made by its manufacturer, is not guaranteed or endorsed by the publisher.

## References

[1] Siegel RL Miller KD Fuchs HE Jemal A. Cancer Statistics, 2021. CA Cancer J Clin (2021) 71:7–33. doi: 10.3322/caac.21654 33433946

[2] Pondé NF Zardavas D Piccart M. Progress in Adjuvant Systemic Therapy for Breast Cancer. Nat Rev Clin Oncol (2019) 16:27–44. doi: 10.1038/s41571-018-0089-9 30206303

[3] Cancer Genome Atlas Network. Comprehensive Molecular Portraits of Human Breast Tumours. Nature (2012) 490:61–70. doi: 10.1038/nature11412 23000897 PMC3465532

[4] Makama M Drukker CA Rutgers EJT Slaets L Cardoso F Rookus MA. Corrigendum to “An Association Study of Established Breast Cancer Reproductive and Lifestyle Risk Factors With Tumour Subtype Defined by the Prognostic 70-Gene Expression Signature (MammaPrint^®^)” [Eur J Cancer 75 (April 2017) 5–13]. Eur J Cancer (2018) 96:131–2. doi: 10.1016/j.ejca.2018.03.017 29656866

[5] Cejalvo JM Pascual T Fernández-Martínez A Brasó-Maristany F Gomis RR Perou CM. Clinical Implications of the non-Luminal Intrinsic Subtypes in Hormone Receptor-Positive Breast Cancer. Cancer Treat Rev (2018) 67:63–70. doi: 10.1016/j.ctrv.2018.04.015 29763779

[6] Elomrani F Zine M L’annaz S Ouziane I Mrabti H Errihani H. Management of Early Breast Cancer in Older Women: From Screening to Treatment. Breast Cancer Targets Ther (2015) 7:165. doi: 10.2147/BCTT.S87125 PMC4500607 26185468

[7] Spoerke JM Gendreau S Walter K Qiu J Wilson TR Savage H. Heterogeneity and Clinical Significance of ESR1 Mutations in ER-Positive Metastatic Breast Cancer Patients Receiving Fulvestrant. Nat Commun (2016) 7:11579. doi: 10.1038/ncomms11579 27174596 PMC4869259

[8] Roundtree IA Evans ME Pan T He C. Dynamic RNA Modifications in Gene Expression Regulation. Cell (2017) 169:1187–200. doi: 10.1016/j.cell.2017.05.045 PMC5657247 28622506

[9] Huang H Weng H Chen J. M6a Modification in Coding and Non-Coding RNAs: Roles and Therapeutic Implications in Cancer. Cancer Cell (2020) 37:270–88. doi: 10.1016/j.ccell.2020.02.004 PMC7141420 32183948

[10] Lee M Kim B Kim VN. Emerging Roles of RNA Modification: M 6 A and U-Tail. Cell (2014) 158:980–7. doi: 10.1016/j.cell.2014.08.005 25171402

[11] Yang Y Hsu PJ Chen Y-S Yang Y-G. Dynamic Transcriptomic M6a Decoration: Writers, Erasers, Readers and Functions in RNA Metabolism. Cell Res (2018) 28:616–24. doi: 10.1038/s41422-018-0040-8 PMC5993786 29789545

[12] Yi Y-C Chen X-Y Zhang J Zhu J-S. Novel Insights Into the Interplay Between M6a Modification and Noncoding RNAs in Cancer. Mol Cancer (2020) 19:121. doi: 10.1186/s12943-020-01233-2 32767982 PMC7412851

[13] Batista PJ Chang HY. Long Noncoding RNAs: Cellular Address Codes in Development and Disease. Cell (2013) 152:1298–307. doi: 10.1016/j.cell.2013.02.012 PMC3651923 23498938

[14] Dai F Wu Y Lu Y An C Zheng X Dai L. Crosstalk Between RNA M6a Modification and Non-Coding RNA Contributes to Cancer Growth and Progression. Mol Ther Nucleic Acids (2020) 22:62–71. doi: 10.1016/j.omtn.2020.08.004 32911345 PMC7486578

[15] Lan Y Liu B Guo H. The Role of M6A Modification in the Regulation of Tumor-Related lncRNAs. Mol Ther Nucleic Acids (2021) 24:768–79. doi: 10.1016/j.omtn.2021.04.002 PMC8094576 33996258

[16] Zuo X Chen Z Gao W Zhang Y Wang J Wang J. M6A-Mediated Upregulation of LINC00958 Increases Lipogenesis and Acts as a Nanotherapeutic Target in Hepatocellular Carcinoma. J Hematol Oncol (2020) 13:5. doi: 10.1186/s13045-019-0839-x 31915027 PMC6951025

[17] Chen S Zhou L Wang Y. ALKBH5-Mediated M6a Demethylation of lncRNA PVT1 Plays an Oncogenic Role in Osteosarcoma. Cancer Cell Int (2020) 20:34. doi: 10.1186/s12935-020-1105-6 32021563 PMC6993345

[18] Wu Y Yang X Chen Z Tian L Jiang G Chen F. M6a-Induced lncRNA RP11 Triggers the Dissemination of Colorectal Cancer Cells. Via Upregulation Zeb1 Mol Cancer (2019) 18:87. doi: 10.1186/s12943-019-1014-2 30979372 PMC6461827

[19] Zhang Y Yang W Li D Yang JY Guan R Yang MQ. Toward the Precision Breast Cancer Survival Prediction Utilizing Combined Whole Genome-Wide Expression and Somatic Mutation Analysis. BMC Med Genomics (2018) 11:104. doi: 10.1186/s12920-018-0419-x 30454048 PMC6245494

[20] Tellez-Gabriel M Knutsen E Perander M. Current Status of Circulating Tumor Cells, Circulating Tumor DNA, and Exosomes in Breast Cancer Liquid Biopsies. Int J Mol Sci (2020) 21:9457. doi: 10.3390/ijms21249457 PMC7763984 33322643

[21] Lan T Li H Zhang D Xu L Liu H Hao X. KIAA1429 Contributes to Liver Cancer Progression Through N6-Methyladenosine-Dependent Post-Transcriptional Modification of GATA3. Mol Cancer (2019) 18:186. doi: 10.1186/s12943-019-1106-z 31856849 PMC6921542

[22] Zhang S Zhao BS Zhou A Lin K Zheng S Lu Z. M 6 A Demethylase ALKBH5 Maintains Tumorigenicity of Glioblastoma Stem-Like Cells by Sustaining FOXM1 Expression and Cell Proliferation Program. Cancer Cell (2017) 31:591–606.e6. doi: 10.1016/j.ccell.2017.02.013 28344040 PMC5427719

[23] Wang X Zhang J Wang Y. Long Noncoding RNA GAS5-AS1 Suppresses Growth and Metastasis of Cervical Cancer by Increasing GAS5 Stability. Am J Transl Res (2019) 11:4909–21. PMC6731424 31497208

[24] Li X Li Y Yu X Jin F. Identification and Validation of Stemness-Related lncRNA Prognostic Signature for Breast Cancer. J Transl Med (2020) 18:331. doi: 10.1186/s12967-020-02497-4 32867770 PMC7461324

[25] Wu Q Li Q Zhu W Zhang X Li H. Identification of Autophagy-Related Long non-Coding RNA Prognostic Signature for Breast Cancer. J Cell Mol Med (2021) 25:4088–98. doi: 10.1111/jcmm.16378 PMC8051719 33694315

[26] Ma W Zhao F Yu X Guan S Suo H Tao Z. Immune-Related lncRNAs as Predictors of Survival in Breast Cancer: A Prognostic Signature. J Transl Med (2020) 18:442. doi: 10.1186/s12967-020-02522-6 33225954 PMC7681988

[27] Li X Jin F Li Y. A Novel Autophagy-Related lncRNA Prognostic Risk Model for Breast Cancer. J Cell Mol Med (2021) 25:4–14. doi: 10.1111/jcmm.15980 33216456 PMC7810925

[28] Xu S-P Zhang J Sui S-Y Bai N-X Gao S Zhang G-W. Downregulation of the Long Noncoding RNA EGOT Correlates With Malignant Status and Poor Prognosis in Breast Cancer. Tumor Biol (2015) 36:9807–12. doi: 10.1007/s13277-015-3746-y 26159853

[29] Nelson MA Ngamcherdtrakul W Luoh S-W Yantasee W. Prognostic and Therapeutic Role of Tumor-Infiltrating Lymphocyte Subtypes in Breast Cancer. Cancer Metastasis Rev (2021) 40:519–36. doi: 10.1007/s10555-021-09968-0 PMC8424653 33963482

[30] Qiu S-Q Waaijer SJH Zwager MC de Vries EGE van der Vegt B Schröder CP. Tumor-Associated Macrophages in Breast Cancer: Innocent Bystander or Important Player? Cancer Treat Rev (2018) 70:178–89. doi: 10.1016/j.ctrv.2018.08.010 30227299

