# Supplementary material for: Identification and Validation of m6A-Related lncRNA Signature as Potential Predictive Biomarkers in Breast Cancer
Source: Front Oncol. 2021 Oct 15;11:745719. doi: 10.3389/fonc.2021.745719 (PMC8555664; doi:10.3389/fonc.2021.745719)
Supplement: Supplementary file 1 [file Table_1.doc]

| Supplementary Table 1. Clinical characteristics of BC patients in the TCGA database | |
| --- | --- |
| **Characteristic** | **BC patients (n = 1053)** |
| **Vital status, n (%)** |  |
| Alive | 911 (86.5) |
| Dead | 142 (13.5) |
| **Age, n (%)** |  |
| < 65 | 730 (69.3) |
| ≥ 65 | 323 (30.7) |
| **WHO-Stage, n (%)** |  |
| Ⅰ | 172 (16.3) |
| Ⅱ | 597 (56.7) |
| Ⅲ | 240 (22.8) |
| Ⅳ | 20 (1.9) |
| X | 13 (1.2) |
| Unknow | 11 (1.1) |
| **AJCC-T stage, n (%)** |  |
| T1 | 270 (25.6) |
| T2 | 614 (58.3) |
| T3 | 129 (12.3) |
| T4 | 37 (3.5) |
| TX | 3 (0.3) |
| **AJCC-N stage, n (%)** |  |
| N0 | 484 (46.0) |
| N1 | 354 (33.6) |
| N2 | 119 (11.3) |
| N3 | 76 (7.2) |
| NX | 20 (1.9) |
| **AJCC-M stage, n (%)** |  |
| M0 | 876 (83.2) |
| M1 | 22 (2.1) |
| MX | 155 (14.7) |
