# Supplementary material for: Identification and Validation of m6A-Related lncRNA Signature as Potential Predictive Biomarkers in Breast Cancer
Source: Front Oncol. 2021 Oct 15;11:745719. doi: 10.3389/fonc.2021.745719 (PMC8555664; doi:10.3389/fonc.2021.745719)
Supplement: Supplementary file 2 [file Table_2.doc]

| Supplementary Table 2. Clinical characteristics of BC patients in the external validation cohort | |
| --- | --- |
| **Characteristic** | **external validation cohort data (n = 20)** |
| **Age, n (%)** |  |
| < 65 | 13 (65) |
| ≥ 65 | 7 (35) |
| **WHO-Stage, n (%)** |  |
| Ⅰ | 5 (25) |
| Ⅱ | 4 (20) |
| Ⅲ | 10 (50) |
| Ⅳ | 1 (5) |
| **AJCC-T stage, n (%)** |  |
| T1 | 5 (25) |
| T2 | 3 (15) |
| T3 | 7 (35) |
| T4 | 5 (25) |
| **AJCC-N stage, n (%)** |  |
| N0 | 4 (20) |
| N1 | 2 (10) |
| N2 | 9 (45) |
| N3 | 5 (25) |
| **AJCC-M stage, n (%)** |  |
| M0 | 19 (95) |
| M1 | 1 (5) |
