# Supplementary material for: Identification and Validation of m6A-Related lncRNA Signature as Potential Predictive Biomarkers in Breast Cancer
Source: Front Oncol. 2021 Oct 15;11:745719. doi: 10.3389/fonc.2021.745719 (PMC8555664; doi:10.3389/fonc.2021.745719)
Supplement: Supplementary file 3 [file Table_3.doc]

| Supplementary Table 3. qPCR primer sequence | | | |
| --- | --- | --- | --- |
| LncRNA | Species | Forward | Reverse |
| Z68871.1 | Human | ACACCAAACCTTGCATCCCT | ATGTCACACGATGTTCAAGCTG |
| AL122010.1 | Human | AGATCGCTTGCCTCCAAGTC | GCACAAGCTGGTCAGGAGAT |
| OTUD6B-AS1 | Human | CAGAGCCGAGTCAGCCATAAA | CCGTTTCAAGAGGAGGCATT |
| AC090948.3 | Human | TGATTTAGGGTCTTCCAGCACC | AGTCAAGGGAAGGTGTGTAGT |
| AL138724.1 | Human | TACAATTATAACCCATGCAGTCAGC | TGCCTGACCAGGAATACACTTT |
| EGOT | Human | AAGTGTCTCTGCTCTGGTGG | CTCAGCAGGGACGACGAATG |
| GAPDH | Human | GGAGCGAGATCCCTCCAAAAT | GGCTGTTGTCATACTTCTCATGG |
