# Supplementary figures and images for: Identification and Validation of m6A-Related lncRNA Signature as Potential Predictive Biomarkers in Breast Cancer
Source: Front Oncol. 2021 Oct 15;11:745719. doi: 10.3389/fonc.2021.745719 (PMC8555664; doi:10.3389/fonc.2021.745719)

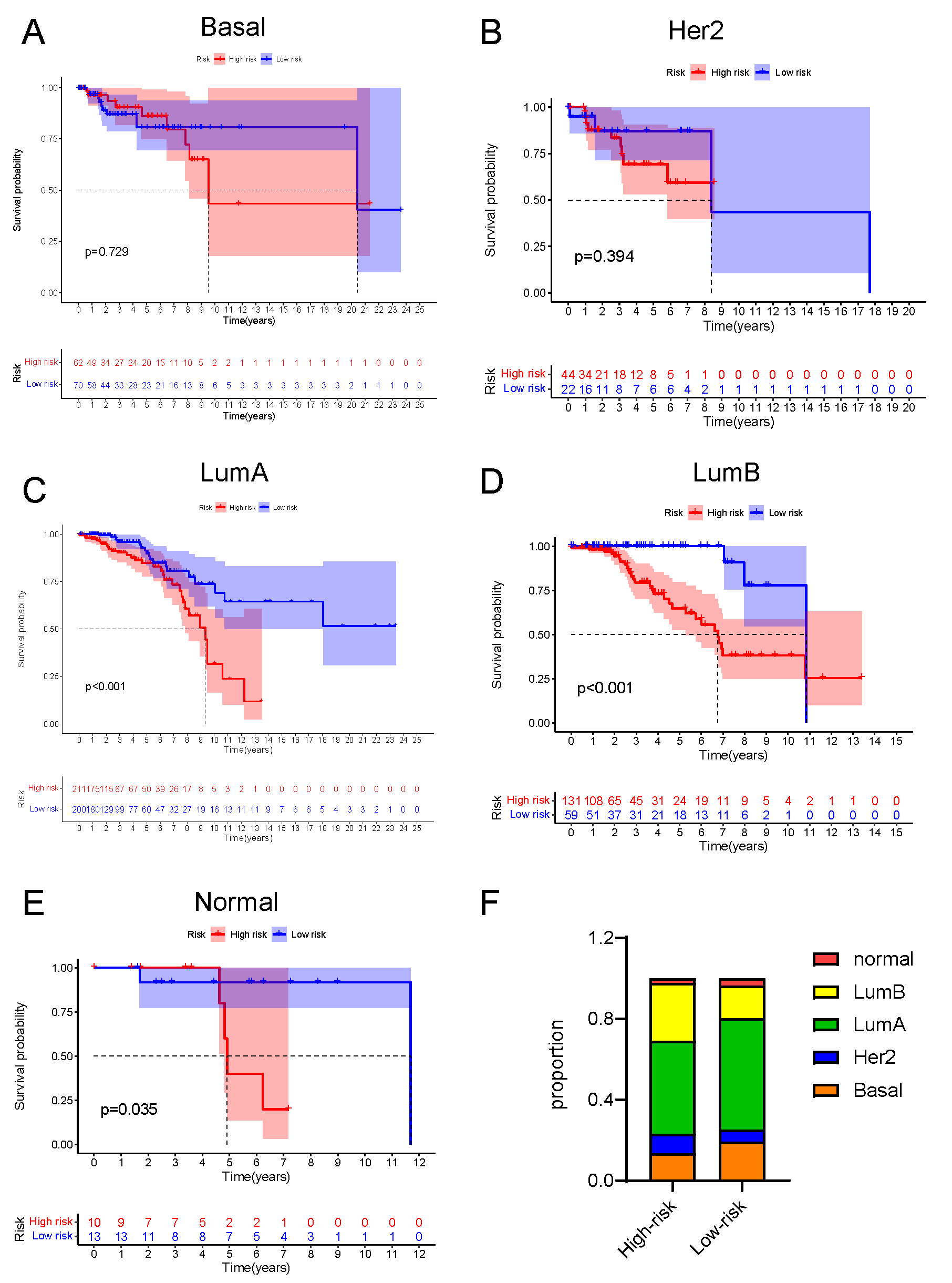

Supplement: Supplementary file 4 [file Image_1.tiff]
